# Unequal risk: Longitudinal observation of socioeconomic disparities and pedestrian mortality patterns in Cali, Colombia

**DOI:** 10.1371/journal.pgph.0006647

**Published:** 2026-08-10

**Authors:** Andrés Villaveces, Lani Fox, Daniel A. Rodriguez, Shrikant I. Bangdiwala, Delia Ortega, Marc Serre

**Affiliations:** 1 Department of Epidemiology, Gillings School of Global Public Health, University of North Carolina at Chapel Hill, Chapel Hill, North Carolina, United States of America; 2 UNC Injury Prevention and Research Center, University of North Carolina at Chapel Hill, Chapel Hill, North Carolina, United States of America; 3 Department of Environmental Sciences and Engineering, Gillings School of Global Public Health, University of North Carolina at Chapel Hill, Chapel Hill, North Carolina, United States of America; 4 Lani Fox Geostatistical Consulting, Claremont, California, United States of America; 5 High Performance Computing and Data Center, University of Alabama, Tuscaloosa, Alabama, United States of America; 6 Department of City and Regional Planning and Institute of Transportation Studies, University of California Berkeley, Berkeley, California, United States of America; 7 Population Health Research Institute, McMaster University, Hamilton, Ontario, Canada; 8 Department of Health Research Methods, Evidence, and Impact, Faculty of Health Sciences, McMaster University, Hamilton, Ontario, Canada; 9 Pontifical Javeriana University, Cali, Colombia; 10 Cisalva Institute, University of El Valle, Cali, Colombia; Yale University School of Medicine, UNITED STATES OF AMERICA

## Abstract

Household locations of pedestrian injuries and fatalities are disproportionately concentrated in lower socioeconomic neighborhoods, reflecting deep-rooted disparities in urban infrastructure, pedestrian exposure, and traffic safety. This study analyzes the relationship between socioeconomic levels and pedestrian incidents in Cali, Colombia, focusing on both the residential locations of injured individuals and the physical locations of fatalities. By separately examining injury residences and fatality locations, we begin to investigate how socioeconomic disparities influence both who is affected and where incidents occur. Residential injury data (2009–2010) and pedestrian fatality data (2008–2010) were analyzed using spatial mapping and Poisson regression modeling. Our preliminary findings reveal individuals injured in pedestrian incidents predominantly reside in lower socioeconomic neighborhoods, where inadequate infrastructure and increased exposure to traffic hazards are likely contributing factors. In contrast, fatal incidents tend to cluster in specific high-risk zones, particularly in middle-high socioeconomic neighborhoods characterized by dangerous road conditions and high vehicle speeds. Poisson regression identified socioeconomic levels and proximity to health centers were significant predictors of pedestrian fatalities, while road and population density showed weaker or nonsignificant associations. These findings provide an initial foundation for hypothesis generation and future research on urban safety disparities. They suggest socioeconomic context and access-related infrastructure together influence pedestrian risk, underscoring the need to integrate equity-focused and built-environment strategies into pedestrian safety planning and policy. By examining both socioeconomic context and physical risk, this study offers early, but critical insights for creating safer, more equitable urban environments.

## Introduction

Globally, road traffic injuries remain a critical public health challenge, responsible for approximately 1.3 million deaths each year [1]. Pedestrians account for a substantial proportion of these fatalities despite ongoing safety interventions [2–4]. In Latin America and countries such as Colombia, pedestrian injuries rank as the second leading cause of transportation deaths [5]. yet analyses integrating socioeconomic and spatial dimensions remain limited. While traditionally viewed primarily through the lens of traffic volume and roadway infrastructure, increasing evidence indicates pedestrian injury and fatality patterns are influenced by socioeconomic inequalities and built-environment conditions [6–10]. One important dimension of this context is socioeconomic level (SEL), which reflects the neighborhood infrastructural context that influence patterns of exposure, the burden of pedestrian injuries, and access to protective safety measures [11–15].

Recognizing pedestrian injury risk is shaped not only by traffic conditions but also by structural and socioeconomic context, movements such as Vision Zero and Safe Systems call for eliminating traffic-related deaths by centering design around the needs of “vulnerable users,” most commonly pedestrians. This framing rests on the logic a system designed to protect those least tolerant of crash impacts will be safe for all users. As walking is not always discretionary, lower-income households are more likely to rely on walking for employment, shopping, or transit access, whereas higher-income households more often engage in flexible, discretionary walking. As a result, exposure to traffic environments is patterned by socioeconomic position, reflecting structural mobility constraints rather than individual choice alone [16,17]. Although Vision Zero and Safe Systems were developed within a U.S. policy context, their perspective resonates with patterns observed in Latin America, where lower socioeconomic neighborhoods exhibit concentrated injury burdens consistent with structurally patterned exposure rather than individual choice alone [18].

Road traffic injury prevention, however, extends beyond infrastructure alone and also reflects the broader safety environment, including driver behavior and the strength and enforcement of traffic safety laws [16,17,19]. Evidence from urban settings suggests adoption of basic safety practices can vary substantially across cities; for example, observational studies of child restraint use demonstrate uneven implementation of occupant protection measures despite existing regulations [19]. A persistent challenge in this field is the absence of detailed exposure measures, which constrains interpretation of observed disparities [15]. A 2025 PAHO/WHO report on Road Traffic Injuries in Latin America and the Caribbean highlighted that, in Colombia, structural inequities continue to drive preventable differences in traffic related mortality [20]. Similarly, Rampinelli et al found socioeconomic and built-environment inequalities jointly predicted pedestrian injuries in Santiago, Chile, underscoring systemic inequities, rather than individual behaviors alone shape risk [21].

Furthermore, despite repeated recognition of the importance of socioeconomic equity in pedestrian safety, empirical research integrating socioeconomic and spatial dimensions remains limited globally, particularly in Latin America [8,18,22]. The present study builds on this perspective by distinguishing between where injured individuals reside and where fatal incidents physically occur in Cali, Colombia, integrating socioeconomic and spatial dimensions to examine how neighborhood context shapes both who is affected and where lethal events are concentrated [23].

Analytically, residential location reflects the socioeconomic and infrastructural conditions shaping daily mobility and exposure patterns, whereas event location reflects the spatial concentration of high-risk environments within the urban landscape. Examining both dimensions allows assessment of whether pedestrian inequities arise primarily from systemic residential disadvantage, localized environmental hazards, or their interaction. The analysis also distinguishes between burden and risk: absolute counts of injuries capture the concentration of affected individuals, while population-standardized fatality rates estimate relative risk per capita.

Cali, Colombia provides a valuable context for examining these disparities due to its high pedestrian fatality rates and significant socioeconomic inequalities [18,24–26]. We aim to provide preliminary evidence guiding future hypothesis generation and equity-focused interventions to reduce pedestrian mortality and morbidity in Latin America and other urban environments worldwide [25,27,28]. Although the data analyzed in this study span 2008–2010, the structural determinants of pedestrian injury and fatality, including infrastructure disparities, mobility dependence, and socioeconomic inequities are persistent features of many urban environments [29,30]. As such, these records provide an important structural baseline, enabling examination of residential vulnerability and spatial clustering prior to more recent shifts in travel behavior and fatality trends. Historical administrative datasets are also valuable for identifying enduring patterns of inequality and contextualizing recent trends in pedestrian injuries and fatalities.

## Methods

This study employs two complementary approaches. First, descriptive spatial mapping of raw injury (residence) and fatality (event location) counts was conducted to visualize absolute burden. Second, a Poisson regression model was used to estimate population-standardized pedestrian fatality risk across neighborhoods, incorporating socioeconomic and built-environment predictors. The regression analysis focused exclusively on pedestrian fatalities (2008–2010), as residence data for non-fatal injuries were available only for 2009–2010 and were not consistently geocoded nor temporally complete. As the time periods differ slightly between datasets (injury residences: 2009–2010; fatality locations: 2008–2010), they were not statistically compared across time. Rather they were analyzed separately to illustrate complementary dimensions of spatial burden and risk, thus the choropleth maps display count-based spatial patterns, whereas regression-based maps estimate relative risk (rates) across the city. Geobiz was utilized for geocoding [31].

Pedestrian fatality and injury data were obtained from official traffic reports recorded in the Cali Injury Mortality Surveillance System and were acquired directly from the Office of the Mayor of Cali through the Secretariat of Mobility. The dataset includes location and date of all transport related fatal injuries in the urban area of Cali from January 2008 through December 2010. Residences of injuries were sourced from hospital records and emergency response reports. Injury cases included pedestrians presenting for medical care or transported via emergency services during 2009–2010. These data primarily reflect medically attended injuries and are therefore more likely to capture moderate to severe cases. Minor injuries not resulting in ambulance dispatch or hospital presentation are unlikely to be represented. Data with residential information included non-fatal injuries and injuries that later resulted in fatalities, while pedestrian fatality data refer only to incidents that directly caused death. The Administrative Department of Municipal Planning of Cali supplied the official census shapefiles for the city (a nontopological file format that stores the geometric location and attribute information of geographic features of Cali, Colombia), containing administrative urban boundaries, built environment variables, population data, and socioeconomic levels for neighborhoods in Cali.

Socioeconomic levels contained in this shapefile were developed by Colombia’s National Administrative Department of Statistics (DANE) and implemented by municipal governments, such as Cali. In Colombia, socioeconomic level is officially categorized into six residential levels (1 = low-low, 2 = low, 3 = medium-low, 4 = medium, 5 = medium-high, and 6 = high), primarily used for taxation and public service subsidy allocation [32]. The classification is based on observable dwelling characteristics (e.g., construction materials, finishes, built area) and neighborhood infrastructure features (e.g., road type, service availability) and is assigned at the neighborhood level. These strata do not correspond to explicit income thresholds or individual-level characteristics such as education or occupation, but instead represent ordered categories of residential quality and neighborhood infrastructure conditions. In general terms, Stratum 1 reflects the lowest levels of housing quality and infrastructure (e.g., incomplete services, unpaved roads, limited finishes, smaller built area), while Strata 5–6 represent high-quality construction, complete services, paved access, superior finishes, and larger residential units. Intermediate strata (2–4) capture gradations in structural condition, service access, and housing amenities [32–34].

A Poisson regression model was then fitted to these population-standardized death location rate estimates, incorporating socioeconomic and selected built-environment predictors. The spatial scale of analysis was determined using a Land Use Regression (LUR) optimization approach [25,26,35]. To reduce small-number instability, incidents were aggregated using a rolling 9-month window prior to modeling. The full Poisson model for fatalties is specified as:

log(*μ*_*i*_) = *β*_*0*_ + *β*_*1*_*X*_*1i*_+*β*_*2*_*X*_*2i*_ + ⋯ + *β*_*k*_*X*_*ki*_*,* where *μ*_*i*_ is the expected pedestrian mortality rate for grid points. *i* and *X*_*ji*_ are the *j*th covariates (including socio economic and environmental predictors), and the *β* coefficients correspond to the log-risk ratios (Table 1 reports IRRs as exp(*β*)). The nineteen spatial variables used in the Poisson regression represent complementary dimensions of the urban environment across three geographic data types: points (e.g., health centers, hydrants, schools, police stations), lines (e.g., primary, secondary, and tertiary roads), and polygons (e.g., population density, sidewalk coverage, open areas, and building density). Features such as hydrants (“hinderance variables”), education centers, and health centers (“mixed use variables”) served as spatial proxies for neighborhood infrastructure density and areas of frequent pedestrian–vehicle interaction following established spatial exposure methods. Additional Details regarding the Poisson Model can be found in S1 Table.

**Table 1:** Summarized statistics of Residential locations of injured pedestrians (2009–2010) and locations of pedestrian deaths (2008–2010) by neighborhood socioeconomic level.

Neighborhood	Residences of Injured (Individual Counts; 2009–2010)			Death Locations (Individual Counts; 2008–2010)		
Socioeconomic Level: Total Category	Total Residents Injured (Absolute Count; % of Total)		Mean Resident Injuries per Neighborhood	Total Deaths (Absolute Count; % of Total)		Mean Deaths per Neighborhood
Very Low: 17	456 (17.7%)		26.8	35 (11.4%)		2.1
Low: 42	882 (34.2%)		21.0	88 (28.7%)		2.1
Middle-Low: 62	992 (38.4%)		16.0	122 (39.7%)		2.0
Middle: 8	105 (4.1%)		13.1	24 (7.8%)		3
Middle-High: 11	136 (5.3%)		12.4	31 (10.1%)		2.8
High: 1	11 (0.43%)		11.0	7 (2.3%)		7
Total Neighborhoods: 141	2582		18.3	307		2.2

Note: Mean per neighborhood values represent average counts per neighborhood within each socio-economic level category and are not population-standardized rates. Additionally, Injuries and deaths that occurred in neighborhoods categorized as “governmental areas” (i.e., with no assigned socioeconomic value) were excluded from the counts presented in the table.

All main results, including statistical significance and effect estimates, are reported from the full multivariable model, which accounts for the joint effects of all covariates. To illustrate the spatial pattern associated with socioeconomic level (SEL), the SEL coefficient from the full multivariable Poisson regression model was used to generate a map of the estimated SEL effect across grid points. This visualization reflects the association between SEL and pedestrian fatality risk while accounting for the other covariates included in the model. The resulting map highlights how the SEL association varies spatially across the city and helps illustrate the geographic distribution of socio-economic disparities in pedestrian fatality risk. This distinction is important for understanding whether observed disparities reflect broader neighborhood conditions, physical environmental hazards, or their interaction. For additional contextual reference, we created an animated time series illustrating our prior geostatistical space–time modeling analysis over the full study period [26]. The visualization includes an inset map of neighborhood socioeconomic levels, enabling examination of how pedestrian mortality risk patterns evolved over time in relation to local SEL. The animation is available online at: at: https://mserre.sph.unc.edu/BMElab_web/mappingStudies/AV-LF_PedMort_Cali_BMELUR_UnequalRisk/index.htm.

MATLAB was utilized to: 1) aggregate pedestrian deaths and injuries based on both the location of the injury or fatality and the residential location of individuals involved, and 2) create regression maps [36]. While ArcGIS was utilized to generate cholopleth maps [37] and Stata was used to conduct the Poisson Regression [38].

## Results

A total of 356 pedestrian deaths were recorded during this period, of which 97% (345/356) were successfully geocoded to a specific address and 307 were located in neighborhoods with assigned socioeconomic levels. A total of 5,451 injured pedestrians were identified in 2009–2010 based on residential address records. Of the total injury records, 3,190 (58.5%) were successfully geocoded to a residential neighborhood. Among these, 2,582 were located in neighborhoods with assigned socioeconomic levels and were included in the residential socioeconomic analysis. Injuries occurring in governmental areas or lacking valid residential geocoding were excluded from the socioeconomic comparison. S1 Table provides additional descriptive information on victim demographics, including age and sex where available, as well as broader demographic context for Cali during the study period.

**Fig 1:**
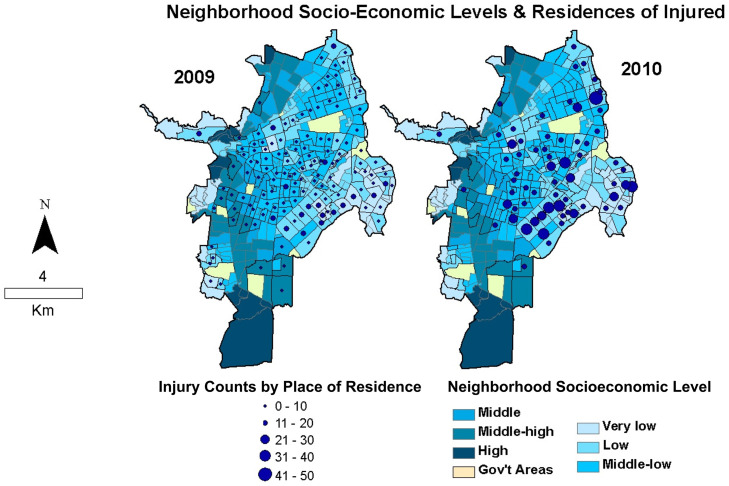
Neighborhood socioeconomic levels and residential locations of injured individuals in Cali, Colombia, for the years 2009 and 2010. Base map/ boundary layer source: Administrative boundary shapefile for Cali, Colombia, provided via the City of Cali and publicly available through the DANE geoportal: https://www.dane.gov.co/files/geoportal-provisional/. Reuse is permitted under DANE’s license and conditions of use: https://geoportal.dane.gov.co/acerca-del-geoportal/licencia-y-condiciones-de-uso/.

**Fig 2:**
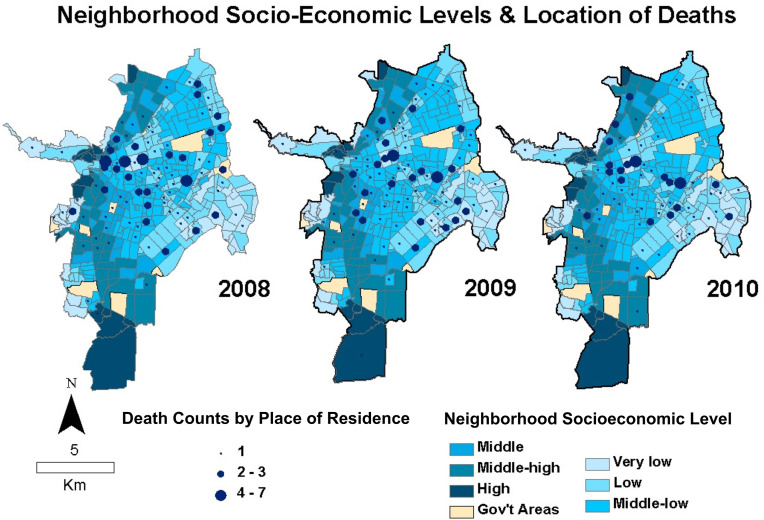
Neighborhood socioeconomic levels and locations of pedestrian fatalities in Cali, Colombia, for the years 2008, 2009, and 2010. Base map/ boundary layer source: Administrative boundary shapefile for Cali, Colombia, provided via the City of Cali and publicly available through the DANE geoportal: https://www.dane.gov.co/files/geoportal-provisional/. Reuse is permitted under DANE’s license and conditions of use: https://geoportal.dane.gov.co/acerca-del-geoportal/licencia-y-condiciones-de-uso/.

Table 1 summarizes the residential locations of injured pedestrians (2009–2010) and the physical locations of pedestrian deaths (2008–2010) across neighborhoods categorized by socioeconomic level (SEL). The table and maps (Figs 1 and 2) present absolute counts of injuries and deaths by neighborhood and SEL. The highest number of injured individuals resided in Middle-Low (992 injuries, 38.4%) and Low-level neighborhoods (882 injuries, 34.2%), together accounting for over 70% of all recorded injuries. Very low-level neighborhoods also contributed an additional 456 incidents (17.7%), while Middle and Middle-High and High neighborhoods recorded fewer than 6% each. When standardized by the number of neighborhoods per SEL, Very Low areas exhibited the highest concentration of injuries (26.8 incidents per neighborhood), followed by Low (21.0) and Middle-Low (16.0). In contrast, the High stratum included only one neighborhood but showed 11 recorded injuries. Pedestrian death locations showed fatalities more evenly distributed across SEL. Middle-Low neighborhoods recorded the highest total number of deaths (122), followed by Low (88) and Middle (31). High neighborhoods reported the highest incidents per neighborhood (7 deaths per neighborhood), despite representing a smaller portion of total neighborhoods; however, it also reported the lowest number of deaths. Injuries and deaths occurring in neighborhoods without assigned socioeconomic values, classified as “governmental areas” (non-residential), were excluded from the counts in the table.

Fig 1 presents a spatial analysis of neighborhood SEL and the residence of injured persons for 2009–2010. Maps highlight disparities in injury patterns based on SEL. Lower socioeconomic neighborhoods: very low, low, and middle-low, are concentrated in the central and eastern parts of the city, while middle-high and high socioeconomic neighborhoods are predominantly located on the city’s periphery. The visual patterns in the maps (Figs 1 and 2) are echoed in Table 1. Middle-low and low-level neighborhoods account for most injuries, (nearly three-quarters of all recorded incidents). Middle-low neighborhoods alone reported nearly 1,000 injuries over the two years (average of 16 injuries per neighborhood). Very low socioeconomic neighborhoods had the highest average number of injuries per neighborhood at 26.8.

Fig 2 displays the spatial distribution of neighborhood socioeconomic levels and the physical locations of deaths across three years: 2008, 2009, and 2010. Data show most deaths occurred in physical locations within middle-low and low-level neighborhoods, reflecting a similar pattern to the residential locations of injured individuals. However, when deaths are analyzed on a per-neighborhood basis, middle-high neighborhoods exhibit the highest average number of deaths per neighborhood, despite having fewer total neighborhoods. In contrast, very low-, low-, and middle-low-income neighborhoods accounted for the highest total death counts but had lower per-neighborhood averages, indicating that fatal incident sites were more widely distributed across multiple neighborhoods.

The Poisson regression models estimate rates of pedestrian fatalities focusing on risk per population, enabling comparisons across neighborhoods of different sizes. Poisson regression results revealed SEL was a significant independent predictor of pedestrian fatality risk: higher SEL was associated with a modest but statistically significant increase in risk (Table 2). Among built environment variables, proximity to health centers had the strongest association with fatality risk, while hydrant and road density were also significant but with smaller effects. Sidewalk density was minimally protective, and population density was not significantly associated with risk. Complete incidence rate ratios (IRRs) and confidence intervals for all predictors are presented in Table 2.

**Table 2:** Poisson regression model results for pedestrian fatality risk, Cali, Colombia (2008–2010).

Variable	IRR	P-value	95% Confidence Interval
Education Centers	1.040	<0.001	(1.021, 1.059)
Health Centers	1.622	<0.001	(1.366, 1.926)
Hydrants	1.093	<0.001	(1.072, 1.115)
Primary and Secondary Roads	1.041	<0.001	(1.026, 1.056)
Sidewalk Density	0.999	0.044	(0.999, 1.000)
Socioeconomic Level	1.066	0.007	(1.018, 1.116)
Population Density	1.00	<0.001	(1.000, 1.000)

**Fig 3:**
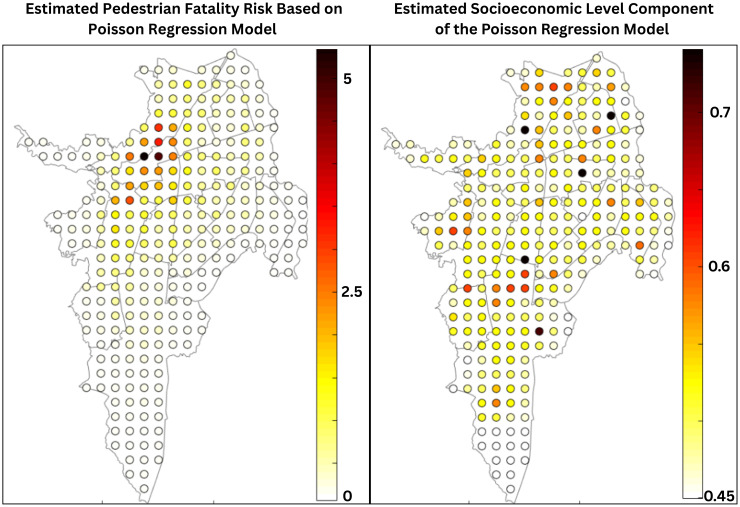
Spatial distribution of estimated pedestrian fatality risk in Cali, Colombia, based on Poisson regression analysis. The left panel (A) shows predicted risk from the full multivariable model incorporating all socioeconomic and built environment predictors. The right panel (B) shows the estimated spatial pattern associated with socioeconomic level (SEL), derived from the SEL coefficient in the full multivariable Poisson regression model while holding the remaining covariates in the model constant. Both maps use the same analysis grid, but color bars are scaled to the range of fitted values in each panel to optimize visualization. Base map/ boundary layer source: Administrative boundary shapefile for Cali, Colombia, provided via the City of Cali and publicly available through the DANE geoportal: https://www.dane.gov.co/files/geoportal-provisional/. Reuse is permitted under DANE’s license and conditions of use: https://geoportal.dane.gov.co/acerca-del-geoportal/licencia-y-condiciones-de-uso/.

Fig 3 presents the estimated pedestrian fatality risk using Poisson regression models applied over a regular analysis grid. Unlike Figs 1 and 2, which display absolute counts of injuries and deaths (burden), Fig 3 depicts population-standardized fatality risk and is therefore not intended for direct comparison with the other two figures. Each grid point represents the predicted fatality rate based on environmental characteristics within an optimized spatial radius determined through the LUR approach. The left panel displays results from the full multivariable Poisson regression model, incorporating all predictors listed in Table 1. Areas of higher estimated risk, indicated by darker colors, are concentrated in regions that likely reflect a combination of factors such as high traffic flow, inadequate pedestrian infrastructure, and socioeconomic disparities.

The right panel displays results from a separately re-fitted univariable Poisson regression model using only average SEL as the predictor, alongside the population offset. This map visualizes the spatial distribution of risk as explained by SEL alone, without accounting for contributions from other covariates. Consequently, the map reflects only the portion of overall risk attributable to SEL and not the total predicted risk across all variables. Areas of higher estimated risk in this model are concentrated primarily in the northern and southwestern parts of the city, which correspond to neighborhoods with higher socioeconomic levels. Regions with lower SEL generally show reduced predicted risk in this univariable model.

Although both maps use the same spatial grid, the risk patterns differ because the right panel reflects only the association with SEL, while the left panel incorporates all predictors in the multivariable model. This association likely reflects the spatial clustering of high-speed arterial roads and major activity centers within certain middle- and higher-SEL districts rather than a protective effect of socio-economic status itself. The color scale in the univariable SEL model is narrower than in the full model, showing a smaller range of predicted rates.

## Discussion

Our analysis highlights the complex relationship between who is affected by pedestrian incidents and where fatal incidents occur by integrating two complementary components: count-based maps illustrating the spatial burden of injuries and deaths, and population-standardized Poisson regression models estimating relative fatality risk across neighborhoods. Based on a three-year dataset from a single city, these findings provide an important foundation for hypothesis generation and the design of larger longitudinal studies exploring how socioeconomic context and built-environment characteristics together shape exposure, burden and risk patterns. By focusing on neighborhood-level socioeconomic characteristics rather than individual attributes, the analysis investigates whether incidents occur in high-risk zones outside victims’ home neighborhoods, suggesting potential structural and exposure-related inequities in the urban environment [23,25].

Colombia’s inequality is deeply spatial, with persistent within-city segregation shaping access to infrastructure and services [21]. The country’s six-level socioeconomic stratification system (estratos), commonly used as a proxy for income groups, reflects these sociospatial divisions [18,24]. Within this context, our preliminary findings indicate pedestrian injuries are concentrated in lower socioeconomic residential areas of Cali, whereas fatalities cluster in more localized high-risk districts, including some middle- and higher-SEL areas that represent a smaller share of the city [6,11]. At first glance, the concentration of injuries in lower socioeconomic neighborhoods may appear inconsistent with the modeled elevation of fatality risk in certain middle- and higher-SEL areas. However, these findings reflect different dimensions of pedestrian safety. Injury maps represent absolute burden (counts), which are influenced by population distribution and mobility dependence, whereas the Poisson regression estimates relative fatality risk per population. Consequently, neighborhoods with high injury counts are not necessarily those with the highest fatality risk per capita.

The elevated fatality risk observed in some higher-SEL areas likely reflects the spatial concentration of high-speed arterial roads, commercial corridors, and through-traffic within urban zones, where built-environment characteristics can increase lethality independent of residents’ socioeconomic status. Concurrently, socioeconomic level remained a significant predictor of fatality risk even after adjusting for built-environment factors such as road density. Together, these findings suggest a dual dynamic: systemic exposure burden in lower-income residential areas, where daily mobility depends heavily on walking and public transportation, and localized environmental hazard in high-traffic districts shaped by urban design that prioritizes vehicle flow over pedestrian safety [9,10,15,30,39,40].

These dynamics complicate simplified notions of the “vulnerable road user,” demonstrating vulnerability may be socially patterned and spatially mobile rather than confined to specific neighborhood types. Consistent with findings across Latin America and the United States, pedestrian crash risk is closely intertwined with broader patterns of racial and income inequality [17,19,24]. Recent U.S. research has also documented shifting crash concentrations toward arterial corridors and neighborhood-scale commercial districts, often in areas with limited automobile access and higher proportions of minority residents, patterns interpreted as structurally patterned exposure linked to necessity-based walking [16,17]. Although observed in a different national and policy context, these findings parallel the patterns identified in Cali, where pedestrian injuries cluster in lower socioeconomic residential areas while fatalities concentrate along high-activity traffic corridors. These findings also align with Vision Zero and Safe Systems principles, which emphasize that traffic deaths are preventable through systemic design changes rather than individual behavior modification. Improving pedestrian safety therefore requires equity-focused planning that accounts for both social vulnerability and environmental hazard [19].

This residence–event location perspective offers insights a LUR analysis of fatality locations alone cannot reveal, as focusing solely on death sites may obscure broader systemic inequities in urban planning and infrastructure investment [30,41]. Although this study cannot evaluate the effectiveness of specific interventions, the observed spatial and socioeconomic patterns suggest different strategies may be appropriate in different contexts. In lower-income neighborhoods where injuries are clustered, investments in pedestrian infrastructure, improved crossings, and safer access to public transportation may help reduce exposure [6,15,42]. In contrast, in middle- and higher-income areas where fatalities are concentrated, traffic-calming measures, speed management, and greater pedestrian–vehicle separation may be more relevant [39,40,43,44].

Model results indicate SEL explains only part of the spatial variation in fatality risk, approximately one-fifth of the highest predicted rates in the full model. Although SEL remained a significant predictor, additional high-risk areas emerged where factors such as proximity to major roads, land use patterns, and population density contributed independently of socioeconomic context. These findings suggest structural and environmental conditions interact with socioeconomic inequality to shape pedestrian risk. Proximity to health centers exhibited the highest incidence rate ratio (IRR = 1.622), indicating higher fatality counts in neighborhoods surrounding these facilities.

These relationships likely reflect higher traffic flow and population density near major activity nodes such as health and commercial hubs rather than direct causal effects. As this is an observational study, these associations should not be interpreted as causal and may instead reflect the location of health care facilities in denser, higher-activity parts of the city. Viewed together, the findings indicate socioeconomic and built-environment factors are interrelated components of pedestrian risk, with fatalities clustering in dense, high-activity areas due to concentrated exposure rather than inherent hazard in any single feature. This distinction is crucial for designing targeted interventions that address both systemic vulnerabilities in low-income neighborhoods and localized risks in high-traffic areas [23,25].

This study has several limitations. First, the analysis relies on two distinct datasets, a) residential locations of injured individuals and b) physical locations of pedestrian fatalities, which cannot be linked at the individual level, limiting the ability to track the full trajectory of incidents from residence to outcome. Second, the datasets differ slightly in temporal coverage, with injury data available for 2009–2010 and fatality data for 2008–2010, which may limit temporal comparability. Third, the regression results estimate independent associations and cannot formally test whether incidence rate ratios differ significantly between predictors. Fourth, built-environment variables represent spatial correlations rather than causal relationships, although many of these structural characteristics change slowly and may serve as reasonable proxies for long-term conditions. Finally, the analysis does not include behavioral, enforcement, or traffic flow measures, such as vehicle speeds, pedestrian behavior, law enforcement intensity, or traffic volumes, which may also influence pedestrian safety outcomes [40,45]. Prior research indicates road safety behaviors are shaped by legal frameworks, enforcement capacity, and social norms, suggesting that factors beyond infrastructure design may also contribute to observed risk patterns [16,19].

In this analysis, socioeconomic level (SEL) was modeled as an ordinal variable, consistent with common practice in spatial and epidemiologic research. Future studies could alternatively treat SEL as categorical to explore potential nonlinearities and estimate category-specific effects. Incorporating direct measures of traffic volume and mobility patterns would also help refine understanding of localized pedestrian risk.

The data analyzed span 2008–2010, one of the few periods in which both residential injury data and geocoded fatality locations were systematically available for Cali. Because the study focuses on a single city, its findings may not be directly generalizable to other urban contexts. However, the purpose of this analysis was not to provide a contemporary surveillance update but to examine structural patterns of pedestrian risk. Persistent factors such as uneven infrastructure investment, differences in pedestrian exposure, and socioeconomic inequality continue to shape pedestrian safety outcomes, making historical datasets valuable for understanding enduring patterns of vulnerability in urban systems [16,17,19]. These findings are consistent with research from other Latin American cities. For example, a geographic analysis in Santiago, Chile (2012–2016) found socioeconomic conditions and built-environment characteristics jointly shape pedestrian injury severity, with severe and fatal crashes concentrated in lower- and middle-income neighborhoods [21]. Together, these studies suggest pedestrian risk reflects systemic disparities in infrastructure and urban design rather than individual behavior alone.

Since the period examined here (2008–2010), urban mobility systems have evolved with the expansion of electric vehicles, micromobility devices, and changes in traffic composition that may alter pedestrian–vehicle interactions. During this time, Cali has also implemented initiatives promoting active transportation, including pedestrian infrastructure investments and programs such as Ciclovía that close major roads to vehicles on Sundays [18]. While this study cannot evaluate the impact of these interventions, the findings provide a baseline for future assessments of pedestrian safety and equity-oriented mobility initiatives.

## Supporting information

S1 Table List of 18 variables used in the analysis, categorized by geographic data. (DOCX)
