# Supplementary material for: Unequal risk: Longitudinal observation of socioeconomic disparities and pedestrian mortality patterns in Cali, Colombia
Source: PLOS Glob Public Health. 2026 Aug 10;6(8):e0006647. doi: 10.1371/journal.pgph.0006647 (PMC13456275; doi:10.1371/journal.pgph.0006647)
Supplement: Supplementary file 1 [file pgph.0006647.s001.docx]

**Supplementary Materials**

Overview

The supplementary materials provide additional methodological detail and supporting information for the spatial and statistical analyses described in the main manuscript. These materials expand on the construction of the spatial modeling framework, the variables included in the analysis, and the implementation of the Poisson regression models used to estimate pedestrian fatality risk across neighborhoods in Cali, Colombia. S1 Table lists the spatial predictors included in the analysis, organized by geographic data type (point, line, and polygon features). Additional sections describe the grid-based spatial framework used to calculate mortality rates, the aggregation procedures applied to reduce small-number instability, and the approach used to assign socioeconomic level and population estimates to circular incidence areas. Supplementary results provide additional descriptive statistics on pedestrian fatalities and injuries, including demographic distributions of victims. Together, these materials provide transparency regarding the analytical procedures used in the study and support reproducibility of the spatial modeling approach.

S1 Table: List of 18 Variables Used in the Analysis, categorized by Geographic Data Type

| **Point** | **Line** | **Polygon** |
| --- | --- | --- |
| Libraries | All Roads | Population Density |
| Police Stations | Primary Roads | Raw Population |
| Fire Hydrants | Secondary Roads | Socio-Economic Status |
| Health Centers | Tertiary Roads |  |
| Educational Centers | Primary and Secondary Roads |  |
| Open Areas/Parks | Sidewalk |  |
| Community Service Centers |  |  |
| Community Centers |  |  |
| Total Lots in Area (Building Density) |  |  |
| Intersections |  |  |

Spatial Modeling Framework and Grid Construction

To link pedestrian mortality to neighborhood socioeconomic levels, a regular grid was created across the urban area. Each grid point served as the centroid of an overlapping circular rate area where death rates, population data, and socioeconomic levels were calculated. Based on prior research on pedestrian fatalities in Cali,[1] 500-meter radius circular incidence areas were implemented, with centroids placed on a regular grid spaced 650 meters apart (2R × 0.65).[1,2] Pedestrian mortality rates defined by cases/population at each grid point were calculated by aggregating all deaths occurring within the surrounding circular incidence area and dividing by the associated population. To reduce small-number instability, incidents included in the Poisson regression were aggregated using a rolling 9-month window. The Poisson models was then fitted to these rate-based estimates, allowing evaluation of spatial patterns in mortality risk relative to neighborhood socioeconomic context. For socioeconomic level, census sector values within each circular incidence area were assigned based on the modal distribution for the neighborhood and rounded to the nearest official socioeconomic categorization.

Calculations and weighting methods used to estimate circular-area populations follow the approach outlined in Fox et al, 2024.[2] Grid-based circular incidence areas used in this analysis are uniform in size and shape, providing a consistent spatial framework across the city. While these grid areas overlap multiple census sectors, population within each was calculated proportionally from observed sector-level data, maintaining alignment with official counts. This design reduces the boundary and scale-related biases that occur when estimating population for irregular administrative units of unequal area. In contrast, neighborhood-level population data were not available, and interpolation across uneven administrative boundaries would have introduced error; therefore, descriptive maps were generated using raw counts rather than population-adjusted rates.

Land Use Regression (LUR) is fit to data aggregated within overlapping circular incidence areas, where each grid centroid’s predictors and outcome reflect the events and covariates in the surrounding 500 m radius. Because these circular areas overlap, adjacent grids inherently share a portion of their underlying data, both in mortality rates and in socioeconomic/environmental predictors. This overlapping design preserves local spatial autocorrelation in both the predictors and outcome, ensuring the LUR models spatial patterns reflective of true neighborhood-level dependencies. By aligning the grid and area sizes with the empirically observed spatial autocorrelation range from our prior Cali analysis,[2] the approach avoids artificially imposing independence, instead allowing the LUR to capture spatially structured risk while reducing noise.

As population distribution varies across socioeconomic levels, with lower-income groups representing a larger proportion of Cali’s residents, the Poisson regression incorporates population and density variables to adjust for differential exposure. This ensures that model estimates represent relative risk standardized to population size rather than raw event counts, while simultaneously accounting for built-environment and socioeconomic dimensions of risk beyond traditional traffic measures.

Demographic Characteristics of Pedestrian Fatalities and Injuries
Males accounted for 254 deaths (73.4%), while females accounted for 92 deaths (26.6%), 10 deaths did not have information on the victim’s gender. Age information was available for 330 cases. Among fatalities with valid age data, more than half (51.2%) occurred among individuals aged 60 years and older. Adults aged 40–59 years accounted for 27.9% of deaths, those aged 18–39 years represented 15.5%, and individuals under 18 years comprised 5.5% of cases. The age distribution was relatively consistent across study years.

Age information was available for both years. Injuries were most common among adults aged 18–39 years (41.0%), followed by those aged 40–59 years (23.7%). Children and adolescents aged 0–17 years accounted for 17.8% of injured individuals, while adults aged 60 years and older comprised 17.5%. Sex information was recorded for 2010 only; among injured individuals in that year (n = 1,718), 67.9% were male and 32.1% were female According to population estimates from **Colombia’s National Administrative Department of Statistics (DANE)**, during the study period Cali maintained a predominantly working-age population structure, with the largest cohorts between approximately 20 and 49 years of age and progressively smaller proportions of children and older adults. Within the pedestrian injury dataset, the majority of injured individuals were concentrated among adults aged **18–39 years**, while fatalities were more frequent among **older adults aged 60 years and above**. This distribution reflects the broader demographic composition of the city, where working-age adults comprise the largest share of residents.[3]

National age distribution data indicate that Colombia has experienced gradual demographic aging since the mid-1980s, with a modest narrowing of the youngest age cohorts and expansion of middle-aged and older groups. However, these changes have occurred progressively over several decades rather than abruptly during the study period (2008–2010), suggesting that broad demographic structure remained relatively stable in the immediate years surrounding the dataset analyzed here. City-level demographic data indicate that Cali has undergone a more pronounced demographic transition than the national average, with a narrowing of younger age cohorts and a growing proportion of middle-aged and older residents between 1985 and 2018. Although this shift reflects urban aging, these changes occurred progressively over several decades rather than abruptly during the study period (2008–2010). While age is an important determinant of pedestrian exposure and injury risk, the gradual nature of demographic change suggests that age structure alone is unlikely to account for the spatial socioeconomic patterns observed in this analysis.[3]

**References**

1. Fox L, Serre ML, Lippmann SJ, Rodriguez DA, Bangdiwala SI, Gutierrez MI, et al. Spatiotemporal approaches to analyzing pedestrian fatalities: the case of Cali, Colombia. Traffic Inj Prev. 2015;16(6):571–7. Epub 2015/01/01. doi: 10.1080/15389588.2014.976336. PubMed PMID: 25551356.
2. Fox LC, Miller WC, Gesink D, Doherty I, Serre ML. Reducing ecological bias and improving insights in sexually transmitted infection outbreak mapping: Syphilis in Forsyth County, a case study. PLOS Computational Biology. 2024. Epub August 2024.
3. **Departamento Administrativo Nacional de Estadística (DANE).** Censo Nacional de Población y Vivienda (CNPV) 2018 [Internet]. Bogotá (Colombia): DANE; 2018 [cited 2026 Mar 9]. Available from: <https://sitios.dane.gov.co/cnpv/#!/>
